# Music Therapy for Older Adults with Dementia: Impact, Challenges and Future Directive in Africa

**DOI:** 10.1002/alz70862_110150

**Published:** 2025-12-23

**Authors:** Bolanle Rebecca Otegbayo

**Affiliations:** ^1^ University College Hospital, Ibadan, Ibadan, Oyo Nigeria

## Abstract

**Background:**

Non‐pharmacological interventions like music therapy are underexplored in dementia care across low‐ and middle‐income countries, including Nigeria. Biomedical approaches dominate, yet they often fail to address the emotional, cognitive, and social needs of older adults with dementia. Music therapy has shown promise as a holistic intervention, but its implementation in Africa faces significant challenges, including limited research, legislative frameworks, and infrastructural support. Addressing these gaps is crucial to improve the quality of life for people living with Alzheimer's disease, behavioral symptoms, and other forms of dementia.

**Method:**

During the COVID‐19 pandemic, the Chief Tony Anenih Geriatrics (CTAG) Centre at the University College Hospital (UCH), Ibadan introduced music therapy to address the limited mobility and activity options available for older adults with dementia. The music therapy intervention was administered over the course of four years, with sessions held four days a week. Each session consisted of culturally relevant musical activities such as singing, dancing, and storytelling, which also promoted physical activity. Feedback was collected from 58 participants through verbal reports and direct observation during the sessions. Caregivers were also interviewed to provide additional insights into the impact of the therapy on the participants' cognitive, emotional, and physical well‐being, including improvements in mood and reduced anxiety.

**Results:**

The program served over a thousand older adults, with feedback showing significant benefits. Familiar indigenous afrocentric music administered evoked positive emotions, reduced anxiety, and alleviated distress. Participants recalled meaningful past experiences, while caregivers reported reduced agitation and overall mood improvements. Group sessions fostered a sense of belonging, joy, and intergenerational connections. Additionally, physical activity, such as dancing and stretching, promoted mobility and overall patient care.

**Conclusion:**

Music therapy is an effective non‐pharmacological intervention for enhancing cognitive, emotional, and physical well‐being in older adults with dementia. Its application in Nigeria has demonstrated significant benefits, particularly when incorporating culturally relevant Afrocentric music. However, challenges such as infrastructural limitations and a lack of legislative support hinder its sustainability and broader implementation. To address these challenges, we are actively advocating for the inclusion of music therapy in national dementia care policies for dementia management in Africa.

